# Mechanical aortic valve without anticoagulation for 33 years in a Yemeni man: a case report

**DOI:** 10.1186/s13256-016-0976-6

**Published:** 2016-06-29

**Authors:** Khadija Aman

**Affiliations:** Faculty of Medicine and Health Sciences, Internal Medicine Department, Aden University, Khormaksar, Aden, Yemen

**Keywords:** Anticoagulant, Aortic prosthesis, Mechanical valve, Antiplatelets, Thrombosis, Embolism

## Abstract

**Background:**

Mechanical prosthetic heart valves have been used for many decades to replace damaged native valves. Guidelines mandate the use of anticoagulant therapy in patients with mechanical prosthetic valves of any type, irrespective of the position in the heart. The rationale for this is to prevent valve thrombosis and thromboembolic complications without increasing the risk of excess bleeding. We report a case involving a patient with a functioning aortic mechanical valve without any anticoagulation therapy for 33 years.

**Case presentation:**

A 46-year-old Yemeni man had an aortic valve replacement, using a St Jude Medical mechanical valve, 33 years ago due to aortic regurgitation grade III–IV of his native valve as a result of rheumatic heart disease. His anticoagulant therapy of Syncumar (acenocoumarol which is a derivative of coumarin) was discontinued 4 months after surgery, and he was sustained on aspirin and digoxin. He presented to our cardiac clinic 33 years later with palpitations, which had started 2 weeks previously. On clinical examination, his condition was fair with a New York Heart Association functional classification of I. He was in sinus rhythm and had normal heart size, as shown on chest X-ray. Echocardiography revealed normal heart chamber dimensions and normal left ventricular systolic and diastolic function. His mean transaortic gradient was 12.58 mmHg and the calculated aortic valve area was 1.44 cm^2^. He was started on anticoagulant therapy.

**Conclusions:**

Only a few cases of well-functioning mechanical valves without the use of anticoagulant therapy for many years have been reported. Our patient is one such case who used only aspirin for 33 years. Further research is needed to understand the interpersonal variations and other unexplored factors in anticoagulant therapy for patients with mechanical prosthetic heart valves.

## Background

Mechanical prosthetic heart valves were first used successfully in 1960 to replace damaged native valves [1]. The valves have the advantage of durability and longevity, but also have many complications, which may lead to failure of valve function. The most common of these complications is the risk of valve thrombosis and systemic thromboembolism. The use of antithrombotic drug therapy is mandatory in all patients with mechanical prosthetic valves. The intensity of treatment should be optimized, without increasing the risk of bleeding [2].

In Yemen, as in most developing countries, the majority of valve replacements are due to destruction of the native valve, caused by rheumatic heart disease [3]. Here, we report the case of a patient who recently presented with palpitations, who had a St Jude Medical aortic valve replacement 33 years previously, with no anticoagulation therapy, treated with only aspirin.

## Case presentation

A 46-year-old Yemeni man presented to our cardiology clinic complaining of occasional episodes of palpitations of 2 weeks’ duration. He had an aortic mechanical valve replacement in Hungary, 33 years ago, at the age of 13, due to aortic regurgitation grade III–IV, with an enlarged left ventricle and impaired systolic function.

After the operation, he was sustained on digoxin, amidopyrine, potassium tablets, and one-quarter of a 10 mg coumarin tablet, and was advised to have continuous anticoagulation therapy (as prescribed in his discharge report). When he arrived in Yemen, 4 months after the operation, he was clinically stable, with no complications, but for unknown reasons, his treatment had been changed by his specialist. Aspirin 75 mg and digoxin were the only drugs used for his therapy for a period of approximately 33 years, until he presented to us with palpitations.

A clinical examination revealed a healthy adult man with a New York Heart Association functional classification of I, a pulse rate of 88 regular beats per minute and blood pressure of 110/80 mmHg. Auscultation of his heart revealed a normal first heart sound and loud mechanical component of the second heart sound and grade II/VI systolic murmur at the base. The rest of the clinical examination was normal.

A chest X-ray revealed a normal-sized heart with cardiothoracic ratio of less than 50 %. Electrocardiography showed a sinus rhythm with no other abnormal changes.

**Fig. 1:**
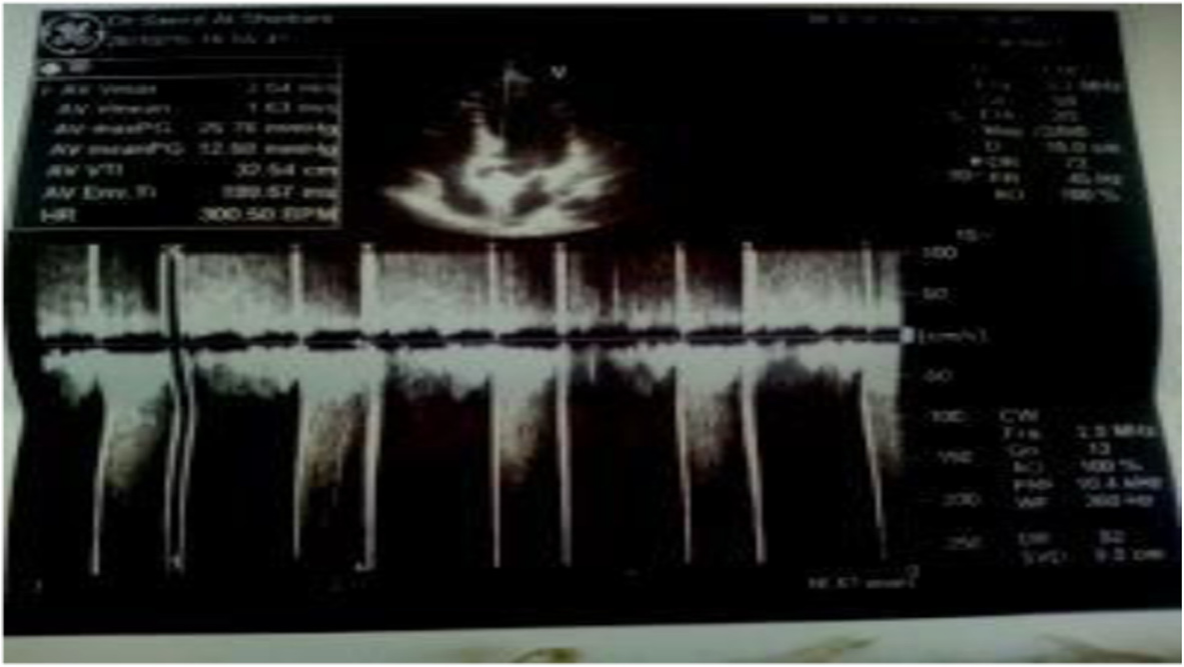
Transthoracic echocardiography of patient’s mechanical prosthetic aortic valve; mean gradient was 12.58 mmHg

Transthoracic echocardiography revealed normal heart chamber size with normal left ventricular systolic and diastolic function. A metallic aortic valve was visualized with peak velocity of 2.54 m/sec and a mean transaortic pressure gradient of 12.58 mmHg (Fig. 1). The aortic valve area was 1.44 cm^2^, and there was normal pulmonary pressure. Due to limitations in the clinical investigation, because of the current war in Yemen, transesophageal echo was not done and fluoroscopy was not available.

Blood tests revealed an international normalized ratio (INR) of 1.0, activated partial thromboplastin time of 30 seconds, hemoglobin level of 124 g/L, and platelet count of 249 × 10^9^/L. His liver, thyroid, and kidney function tests were all normal.

## Discussion

Since the start of heart valve replacement by a mechanical prosthesis, guidelines mandate the use of anticoagulation therapy to prevent valve thrombosis and thromboembolic complications [2, 4]. The prevention of these complications not only depends on effective antithrombotic treatment, but there are many other complex factors which must be taken into consideration, including the surgical procedure itself, amount of time from surgery, type and site of valve device, the number of implanted mechanical prostheses, in addition to the individual risk factors [5].

The majority of thromboembolic complications occur within the first 6 months after surgery. A small number of patients may develop thromboembolic events despite receiving therapeutic anticoagulation. Investigation into the source of the emboli, other than mechanical valve thrombus itself, should be sought [4].

A few cases have reported aortic mechanical valves of any prosthesis type being durable without the use of anticoagulant therapy for long periods of time [6–8], and one case was also reported of a pulmonary valve mechanical prosthesis [9]. In a 25-year follow-up study of patients with a St Jude Medical aortic mechanical prosthesis, valve thrombosis presented in only 0.5 % and thromboembolic event in only 3 % of cases [10].

Abnormal flow across the mechanical valve usually produces zones of low flow, but areas of high shear stress may also be created, leading to platelet activation [11]. Stagnation at the downstream site of the mechanical valve leads to platelet and clotting factor activation [5]. The development of pure carbon technology and On-X mechanical prosthetic heart valves has led to the improvement of blood hemodynamicity, and decreased thromboembolic rates in the absence, or reduced use, of anticoagulation therapy [12]. Previous studies have shown that the risk of thromboembolism is low in children with mechanical bi-leaflet aortic valve replacement, even when treated with antiplatelets alone and no anticoagulant therapy [13, 14]. However, current guidelines still recommend the use of anticoagulants in children as well as in adults [2].

Four months after his operation, our patient, who was 13-years old at the time, stopped anticoagulant therapy, which was replaced by aspirin. In addition, he remained on digoxin for 33 years. An obstructed mechanical valve, caused by a thrombus, is one of the major complications seen in valve replacements, with a rate of 86 % being seen in one study carried out in Yemen [3].

However, we report a case of eventless St Jude Medical mechanical aortic prosthesis of 25 mm size without any anticoagulation therapy, only aspirin 75 mg daily and digoxin for 33 years. Recently, this case presented to us with a functioning valve, but with a 2-week history of palpitations. Although long-term anticoagulant therapy is mandatory for well-functioning mechanical heart prosthesis with no complications of valve thrombosis, only a few exceptions have been reported in the literature [6–9]. How these patients’ valves were protected for such long periods is unknown. It is unknown how our patient’s mechanical valve was protected against thrombosis and thromboembolism, despite receiving only aspirin and digoxin for 33 years, but no anticoagulation therapy.

## Conclusions

It is unusual for a mechanical heart valve to function long term without anticoagulation therapy, using aspirin only. Our patient is now 46-years old, and as he still had a risk of mechanical valve thrombosis he underwent a work up of blood hemostatic function; he was started on oral anticoagulation therapy after discussing with him the risks of living with a prosthetic mechanical heart valve.
